# HydroNeuro: A Data-Efficient IoT Sensing and Edge-AI Framework for Real-Time Hydraulic Anomaly Detection

**DOI:** 10.3390/s26103010

**Published:** 2026-05-10

**Authors:** Nasreddine Somaali, Mohamed Hayouni, Lokman Sboui, Fethi Choubani

**Affiliations:** 1 InnovCom Laboratory, Higher School of Communications (SUP’COM), University of Carthage, Tunis 1054, Tunisia; mohamed.hayouni@supcom.tn (M.H.); fethi.choubani@supcom.tn (F.C.); 2 Institut Supérieur d’Informatique du Kef, Université de Jendouba, Jendouba 8189, Tunisia; 3 Systems Engineering Department, École de Technologie Supérieure (ÉTS), University of Québec, Montréal, QC H3C 1K3, Canada; lokman.sboui@etsmtl.ca

**Keywords:** offline edge AI, hydraulic networks, anomaly detection, neural networks, fractional factorial design, IoT in agriculture

## Abstract

Reliable monitoring of hydraulic networks is essential for efficient and sustainable water management in agriculture. To address the growing need for intelligent, low-latency anomaly detection in such systems, we propose HydroNeuro, a domain-aware embedded framework that integrates hydraulic domain knowledge with data-driven neural inference for the real-time detection of leaks and obstructions. Rather than embedding physical equations directly into the learning objective, we leverage established hydraulic principles, including Bernoulli’s equation and the Darcy–Weisbach formulation, to structure the experimental design, interpret pressure–flow relationships, and ensure physical consistency of the learned representations. These principles confirm that pressure deviations induced by leaks or obstructions are causally explainable and measurable. We employ a fractional factorial design (FFD) to optimize valve activation combinations and sensor configurations during dataset acquisition, thereby reducing redundant experiments, water circulation, and energy consumption while limiting mechanical stress on system components. We deploy a lightweight neural network on an ESP32 microcontroller using TensorFlow Lite for Microcontrollers to enable energy-efficient, low-latency edge inference under severe hardware constraints. Our experimental validation on a laboratory-scale hydraulic testbed demonstrates anomaly detection accuracy exceeding 96%, with strong robustness under sensor noise and hydraulic perturbations. Compared to a multiple linear regression baseline, the proposed neural model reduces the prediction error from an RMSE of 0.58 to 0.12. By coupling physically consistent experimental modeling with embedded neural inference, HydroNeuro provides a scalable and practically deployable solution for autonomous hydraulic monitoring in precision irrigation and smart water distribution systems.

## 1. Introduction

Modern agriculture increasingly relies on engineered hydraulic networks to distribute water efficiently across cultivation areas. In this context, a *hydraulic network* refers to an interconnected system comprising water reservoirs, pumps, pipelines, valves, and pressure or flow sensors designed to regulate and deliver water to multiple irrigation zones. Such infrastructures range from traditional multi-sector drip irrigation systems to hydroponic installations, and collectively account for a substantial share of global freshwater consumption.

Inefficiencies in these networks can lead to water losses exceeding 40–50% [1,2,3,4]. In practical irrigation scenarios, these losses correspond to water that does not effectively contribute to crop uptake, including leakages in distribution pipes, excessive evaporation during surface application, deep percolation beyond the root zone, runoff caused by pressure mismanagement, and non-uniform flow distribution across irrigation sectors. These inefficiencies are particularly critical in rural and semi-arid regions, where limited connectivity and the absence of real-time monitoring systems complicate sustainable water management [3,5,6]. Ensuring food security while minimizing environmental impact therefore requires intelligent and resource-efficient mechanisms capable of continuously monitoring and optimizing hydraulic network performance.

Traditional monitoring approaches, whether manual inspection or semi-automated telemetry, remain inadequate for modern agricultural demands. Such systems are often cost-prohibitive for small-scale deployments, lack real-time responsiveness, and depend heavily on stable Internet connectivity [4,6]. Furthermore, the scarcity of representative hydraulic datasets and the computational limitations of embedded hardware significantly constrain the practical deployment of machine learning algorithms in rural environments [7,8].

Recent advances in IoT-based irrigation systems have improved data acquisition and remote supervision capabilities. Mansoor et al. (2025) reviewed smart sensing technologies for precision agriculture, highlighting connectivity and processing challenges in remote areas [9]. Morchid et al. (2024) proposed a telemetry-based irrigation management system, yet without explicitly addressing hydraulic anomalies such as pressure drops caused by leaks or obstructions [1,2,4]. In parallel, machine learning has been employed for anomaly detection in agricultural and industrial settings. Ileri et al. (2024) introduced a hybrid feature-selection method for IoT-based smart farming networks, focusing primarily on communication data rather than hydraulic dynamics [7]. Similar approaches have been applied to agricultural machinery via CAN-bus analysis [10], while cloud–edge collaborative frameworks have been proposed for distributed anomaly detection [11]. More broadly, recent surveys emphasize the integration of physics-based modeling with machine learning to enhance robustness and interpretability in data-driven systems [12]. Recent studies have also demonstrated the effectiveness of neural networks for internal leakage detection in hydraulic actuator cylinders of heavy machinery, achieving efficient fault classification through supervised learning on pressure and vibration signals [13]. However, these studies rarely incorporate hydraulic behavior modeling or fully embedded deployment pipelines tailored to resource-constrained irrigation infrastructures [8,14].

Edge Artificial Intelligence (Edge-AI) has recently emerged as a promising paradigm for enabling on-device inference, thereby reducing latency, energy consumption, and dependence on cloud connectivity critical requirements in rural agricultural applications [15,16]. Although preliminary demonstrations of embedded anomaly detection exist [14,17], they remain limited in scope, often targeting specific crops, simplified configurations, or partial system implementations.

Despite this progress, significant research gaps persist. Existing studies predominantly focus on irrigation scheduling or water quality monitoring rather than real-time detection of hydraulic anomalies such as leaks and blockages in multi-valve networks. Moreover, few frameworks address the complete embedded workflow, from structured dataset acquisition and model training to TensorFlow Lite conversion, over-the-air updates, and real-time inference on microcontrollers such as the ESP32. Finally, limited adaptability across heterogeneous infrastructures—including traditional irrigation, hydroponic systems, and multi-sector drip networks—restricts scalability in low-connectivity environments. As a result, a fully autonomous, low-cost, and hydraulically grounded embedded system for real-time anomaly detection remains largely unexplored.

To address these challenges, we propose **HydroNeuro**, a physically grounded Edge-AI framework for real-time detection of leaks and obstructions in agricultural hydraulic networks. Rather than embedding fluid dynamic equations directly into the neural loss function, HydroNeuro leverages established principles from fluid mechanics—such as Bernoulli’s equation and the Darcy–Weisbach formulation—to guide the experimental design and interpretation of pressure variations within the hydraulic network. This approach aligns with the broader paradigm of physics-informed machine learning, where physical laws guide data-driven modeling strategies [18]. The framework integrates fractional factorial design (FFD) for resource-efficient dataset generation, lightweight neural modeling supported by universal approximation theory, and full embedded deployment using TensorFlow Lite for Microcontrollers on an ESP32 platform.

In many real-world hydraulic and agricultural infrastructures, collecting large-scale datasets is costly, time-consuming, and sometimes environmentally undesirable due to water consumption and mechanical stress on the infrastructure. Traditional data-driven approaches often assume the availability of large training datasets, which is rarely realistic in operational irrigation networks.

In this work, we address this challenge by combining domain knowledge in hydraulics with an optimized experimental design strategy. Instead of collecting large volumes of redundant data, we employ a fractional factorial design (FFD) to strategically explore the space of valve configurations and flow conditions. This allows us to generate an informative dataset while significantly reducing the number of required experiments.

This approach enables rapid dataset acquisition and real-time training of an embedded AI model capable of reliable anomaly detection despite limited data availability.

The main contributions of this paper are summarized as follows:

- We propose a domain-aware Edge-AI framework that combines hydraulic domain knowledge with neural inference for the real-time detection of leaks and obstructions in irrigation networks, while maintaining physically consistent interpretation of pressure variations.
- We introduce an optimized experimental data acquisition strategy based on fractional factorial design and Hamming weight-driven configuration selection, enabling efficient exploration of nonlinear hydraulic interactions while minimizing the number of required experiments.
- We present an end-to-end embedded deployment pipeline by implementing a lightweight multilayer perceptron (MLP) using TensorFlow Lite for Microcontrollers on an ESP32 platform, enabling low-latency anomaly detection without cloud connectivity.
- We experimentally validate the proposed approach on a laboratory-scale agricultural irrigation testbed, achieving a classification accuracy exceeding 96% while reducing the root-mean-square error (RMSE) from 0.58 (linear baseline) to 0.12.

The remainder of this paper is organized as follows. Section 2 presents the system model and formulates the hydraulic pressure prediction problem. Section 3 describes the proposed HydroNeuro framework, including the physical foundations, the experimental design strategy, and the neural network-based predictive modeling approach. Section 4 details the embedded AI pipeline and system implementation. Section 5 presents the experimental setup and validation results obtained from the HydroNeuro prototype. Section 6 discusses the performance and practical implications of the proposed framework. Finally, Section 7 concludes the paper and outlines potential directions for future work.

## 2. System Model and Proposed Methodology

### 2.1. System Model

**Figure 1:**
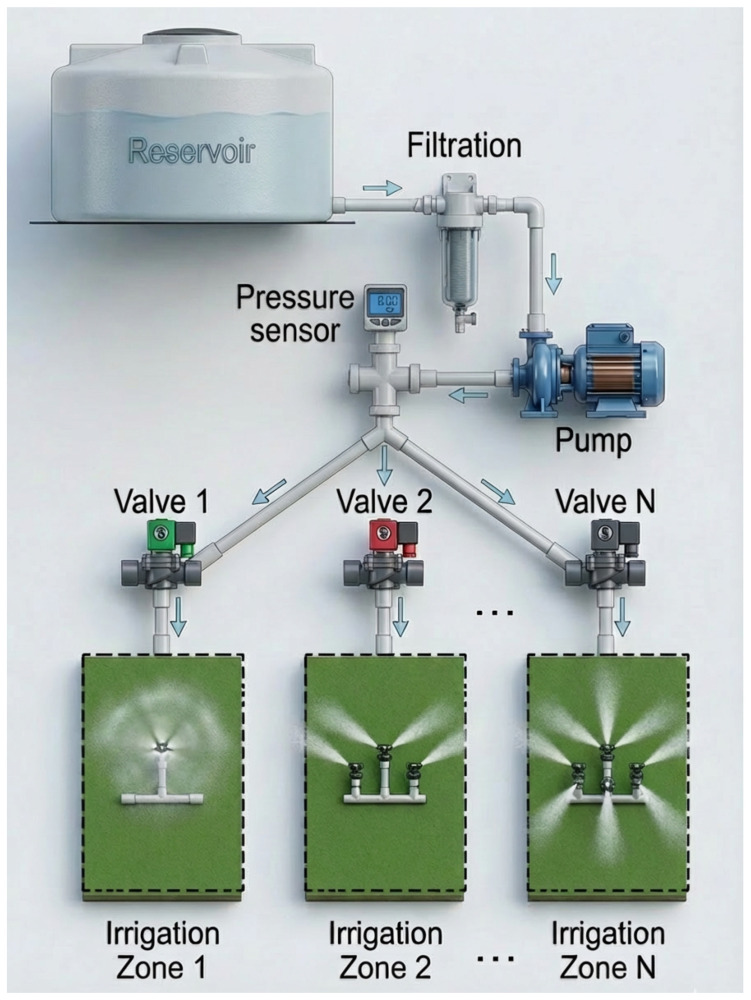
Baseline hydraulic system model considered in this study prior to AI integration.

We consider a pressurized agricultural irrigation network composed of a water reservoir, a pump, *N* electrically actuated valves, interconnected pipe segments, and a distributed pressure sensing node, as shown in Figure 1. The system operates under steady-state incompressible flow conditions, where pressure variations arise from valve activation patterns, hydraulic head losses, and potential anomalies such as leaks or obstructions.

Each valve state is represented as a binary variable $V_{i}\in \left\{0,1\right\}$, where $V_{i}=1$ indicates an open valve and $V_{i}=0$ a closed state. Let $V=\{V_{1},V_{2},\ldots ,V_{N}\}$ denote the valve activation vector, and $P_{m}$ the measured steady-state pressure at the monitoring node. Under normal operating conditions, pressure behavior follows deterministic hydraulic laws, while structural faults such as leakages or partial blockages introduce measurable deviations in pressure distribution.

The objective of the system is to detect anomalies by learning a mapping

$$f:V\to {\hat{P}}_{r}$$

that predicts the reference pressure ${\hat{P}}_{r}$, which is then compared with the measured pressure $P_{m}$ to identify deviations indicative of hydraulic anomalies.

#### Learning Objective

The hydraulic pressure prediction problem is formulated as a nonlinear regression task. The objective is to learn a parametric approximation ${\hat{f}}_{\theta }\left(V\right)$ of the unknown hydraulic mapping such that

(1) $${\hat{f}}_{\theta }=\arg\min_{\theta }E\left[{\left(P_{m}-{\hat{f}}_{\theta }\left(V\right)\right)}^{2}\right]$$

where θ denotes the model parameters and the expectation is approximated empirically over the experimental dataset.

## 3. Proposed HydroNeuro Framework

The HydroNeuro framework adopts a rigorous scientific methodology, combining knowledge of fluid mechanics, statistical design of experiments, and neural network modeling to enable intelligent and resource-efficient management of agricultural hydraulic networks. The neural network itself is purely data-driven and does not embed physical equations.

### 3.1. Physical Basis for Pressure Dynamics

Hydraulic reference pressures ($P_{r}$) can be described using classical fluid mechanics, where Bernoulli’s principle [19] expresses the conservation of mechanical energy in an incompressible, non-viscous flow:

(2) $$P+\rho gh+\frac{1}{2}\rho v^{2}=\mathrm{constant}$$

Here, *P* is the static pressure, ρ the fluid density, *g* the gravitational acceleration, *h* the elevation, and *v* the flow velocity. In irrigation networks, pressure variations are primarily influenced by valve operations and local head losses, which can be approximated using the Darcy–Weisbach equation [20]:

(3) $$\Delta P=f\frac{L}{D}\frac{\rho v^{2}}{2}$$

In Equation (3), *L* denotes the length of the pipe section and *D* the internal diameter.

The physical equations are used only to interpret pressure deviations and not embedded in the neural network. HydroNeuro operates as a purely data-driven black-box predictor rather than a physics-informed neural network (PINN). The role of Bernoulli’s and Darcy’s laws is to provide a scientific explanation: the observed pressure deviations are physically consistent with known hydraulic phenomena, such as leaks, obstructions, or valve-induced flow changes.

Recovering all parameters required for deterministic hydraulic modeling (such as pipe roughness coefficients, precise internal diameters, elevation profiles, junction losses, and boundary conditions) is often impractical in real-world irrigation networks. In many small-scale installations, these parameters are simply not documented: as no built drawings exist, subsequent modifications are not recorded, and maintenance operations (pipe replacements, extensions, valve additions) are carried out without updating any technical records. In other words, the hydraulic identity of the network is largely undocumented. Consequently, the actual physical characteristics frequently diverge from nominal specifications, rendering deterministic modeling unreliable. This situation is particularly common in small-scale or incrementally modified irrigation systems across many regions of Tunisia.

In this context, classical hydraulic principles serve as justificatory and explanatory tools, ensuring that the anomalies detected by the network are physically meaningful. This approach preserves the flexibility, scalability, and practical applicability of the data-driven framework, while grounding it in well-established physics.

By clearly separating physical justification from data-driven inference, HydroNeuro maintains both scientific credibility and operational relevance for real-world agricultural deployments.

### 3.2. Combinatorial Constraints and Fractional Factorial Design

While classical hydraulic theory explains the physical origin of pressure variations, translating these principles into a data-driven learning framework requires systematic experimental observation of valve interaction effects. However, as the number of controllable elements increases, the combinatorial growth of possible operating states rapidly becomes impractical. This scalability constraint motivates the adoption of a structured experimental strategy capable of efficiently capturing dominant hydraulic behaviors without exhaustive enumeration.

For a network with *N* valves, all possible activation states result in $2^{N}$ experimental configurations, which is computationally and materially prohibitive for embedded hydraulic systems. To address this limitation, HydroNeuro adopts a Resolution III fractional factorial design (FFD), reducing the number of required experiments to $2^{N-1}$ in our case N = 4 while preserving the most influential main effects and selected two-factor interactions [21,22].

The resulting pressure response model is expressed as follows:

(4) $$X=\mu +\sum_{i=1}^{N}\beta_{i}V_{i}+\sum_{i<j}\beta_{ij}V_{i}V_{j}+\varepsilon$$

where *X* denotes the measured nodal pressure at the HydroNeuro monitoring point, $V_{i}\in \left\{0,1\right\}$ represents the binary activation state of valve *i*, $\beta_{i}$ and $\beta_{ij}$ correspond to the estimated main and interaction effects, respectively, and ε captures experimental noise and unmodeled hydraulic dynamics.

The statistical significance of each factor is assessed using Analysis of Variance (ANOVA), while model coefficients are estimated through the Ordinary Least Squares (OLS) estimator. ANOVA evaluates the contribution of individual factors and interactions by comparing variance components, whereas OLS minimizes the sum of squared residuals under the Gauss–Markov assumptions, providing unbiased and efficient coefficient estimates.

Receiver Operating Characteristic (ROC) analysis, introduced later for anomaly detection threshold selection, balances sensitivity and specificity by analyzing true positive and false positive rates across varying decision thresholds.

Overall, the adoption of FFD enables systematic exploration of hydraulic network dynamics while significantly reducing experimental burden, water consumption, energy usage, and operational time. This structured design strategy aligns the proposed methodology with sustainable and resource-efficient agricultural practices [1,4].

### 3.3. Data Collection Minimization Strategy

A central objective of this work is to minimize the amount of physical data required to train a reliable anomaly detection model. To minimize experimental effort, we adopt a data-efficient strategy based on fractional factorial design (FFD). Instead of exhaustively exploring all possible valve combinations, the FFD approach selects a subset of configurations that maximizes the statistical information captured by the experiments.

This approach provides several advantages:

- Reduction in experimental time;
- Minimization of water usage during testing;
- Reduction in mechanical wear on hydraulic components;
- Generation of an informative dataset suitable for training lightweight AI models.

As a result, the dataset used for training the neural model can be collected rapidly while preserving the diversity required for reliable anomaly detection.

### 3.4. Hamming Weight-Based Combination Selection

While the fractional factorial design efficiently reduces the number of experimental configurations, it may still miss certain nonlinear interactions that can be critical for the neural network model performance. To address this, we extended the FFD selection using a Hamming weight-based criterion, which prioritizes configurations with a balanced number of valve activations.

For the N=4 valve network, the initial FFD of resolution III provided $2^{N-1}=8$ configurations. By evaluating the Hamming weight of each possible combination, we identified 4 additional configurations that maximized the diversity of binary patterns, resulting in a total of 13 configurations (12 from the fractional factorial and Hamming weight stages, plus 1 empirically validated high-activation boundary.) We measured each configuration three times, ensuring statistical reliability and generating a richer dataset for training the HydroNeuro AI model.

The Hamming weight of a binary activation pattern is defined as the number of bits equal to one (i.e., the number of valves open in a given configuration), and can be mathematically expressed as follows:

(5) $$HW\left(Y\right)=\sum_{i=1}^{N}Y_{i}$$

where $Y=[Y_{1},Y_{2},\ldots ,Y_{N}]$ represents the binary activation vector of *N* valves ($Y_{i}=1$ if valve *i* is open, or 0 otherwise), and HW(Y) is the Hamming weight of the configuration. This concept, widely used in coding and combinatorial design to measure the distribution and diversity of binary codewords [23], provides a principled way to select additional valve combinations that enrich the experimental dataset. To formally ensure diversity across binary activation patterns, the selected configurations maximize the dispersion of Hamming weights:

(6) $$\max_{S}\mathrm{Var}\left(HW\left(Y\right)\right),\;Y\in S$$

where S denotes the selected subset of configurations.

By including configurations with varied Hamming weights, the neural model is exposed to valve patterns with both few and many open valves, enhancing its capacity to model nonlinear interactions in the hydraulic network. This Hamming weight-guided augmentation enhances the representativeness of the experimental space, enabling the neural network to better capture subtle nonlinear interactions between valves. Consequently, the AI model achieves improved predictive accuracy and generalization, especially for rare or intermediate valve activation patterns that would otherwise be underrepresented in a purely FFD-based dataset.

### 3.5. Data Augmentation and Statistical Robustness

HydroNeuro is trained on pressure readings from a single sensor in the irrigation network, together with the corresponding valve states and control signals (ON/OFF). These measurements serve as the primary input to the neural network, enabling detection of anomalies such as leaks or obstructions and prediction of pressure variations under different valve configurations.

In real-world agricultural deployments, collecting exhaustive data for all possible operating conditions is impractical. Sensor measurements naturally contain noise, and certain anomalies occur rarely. Moreover, in practice, acquiring large volumes of field data is challenging due to operational constraints, such as water consumption during testing, energy usage, and wear on pumps and other hydraulic equipment. To address these challenges, HydroNeuro applies a Gaussian-based data augmentation strategy [24]:

(7) $$P_{r}^{\mathrm{aug}}=P_{r}+N\left(0,\sigma^{2}\right)$$

where $P_{r}$ denotes the measured pressure and σ represents the standard deviation of the sensor noise. This augmentation simulates realistic measurement variability and minor hydraulic perturbations, improving the network’s ability to generalize to unseen conditions.

The main objectives of this augmentation are:

To increase the statistical robustness of the model against sensor noise;

To improve generalization for anomaly detection under rare or uncertain operating scenarios.

Additionally, cross-validation and Monte Carlo simulations are used to assess model stability and ensure consistent performance under the stochastic fluctuations typical of agricultural hydraulic networks.

### 3.6. Predictive Modeling

Building upon the pressure dynamics described by (2) and the Darcy–Weisbach loss model in Equation (3), combined with the statistically optimized experimental formulation and Gaussian-augmented dataset, the HydroNeuro framework proceeds to predictive modeling, enabling the identification of abnormal deviations associated with leaks or obstructions.

#### 3.6.1. Linear Regression Baseline

We first implement a multiple linear regression (MLR) model as a baseline to capture the linear relationship between valve states and network pressure:

(8) $${\hat{P}}_{r}=\beta_{0}+\sum_{i=1}^{N}\beta_{i}V_{i}$$

In this equation, ${\hat{P}}_{r}$ denotes the predicted reference pressure at the monitoring node, $V_{i}$ represents the binary activation state of valve *i*, $\beta_{0}$ is the intercept accounting for the baseline network pressure, and $\beta_{i}$ are the regression coefficients quantifying the contribution of each valve to the pressure variations.

We estimate the regression coefficients using the Ordinary Least Squares (OLS) method, which satisfies the Gauss–Markov theorem [25], ensuring unbiased and efficient predictions under standard linearity assumptions.

To detect anomalies, we compare the predicted pressure ${\hat{P}}_{r}$ with the measured pressure $P_{m}$:

(9) $$A=\left\{\begin{array}{cc} 0, & \mathrm{if}\;|{\hat{P}}_{r}-P_{m}|<\varepsilon \\ 1, & \mathrm{otherwise} \end{array}\right.$$

Here, *A* denotes the anomaly flag. An anomaly (A=1) corresponds to a leak when $P_{m}<{\hat{P}}_{r}$ and to an obstruction when $P_{m}>{\hat{P}}_{r}$. The hat symbol in ${\hat{P}}_{r}$ indicates that this is an estimated quantity inferred from data rather than a directly measured physical variable.

We select linear regression as the baseline because it is simple, computationally lightweight, and interpretable. These properties make it suitable for deployment on resource-constrained microcontrollers commonly used in small-scale irrigation systems, where more complex models may be impractical. Moreover, MLR provides a transparent benchmark to evaluate the added value of advanced machine learning methods such as HydroNeuro.

#### 3.6.2. Neural Network Model

To capture nonlinear relationships and environmental disturbances that cannot be modeled by linear approaches, we employ a multilayer perceptron (MLP):

(10) $$h^{\left(l+1\right)}=\phi \left(W^{\left(l\right)}h^{\left(l\right)}+b^{\left(l\right)}\right)$$

In this formulation, the index *l* refers to the layer number within the network architecture. The activation vector of layer *l* is denoted by $h^{\left(l\right)}$, $W^{\left(l\right)}$ is the weight matrix connecting layer *l* to layer l+1, $b^{\left(l\right)}$ represents the corresponding bias vector, and ϕ(·) is a nonlinear activation function applied element-wise. Following the Universal Approximation Theorem [26], such architectures can approximate any continuous function, enabling the model to represent complex hydraulic nonlinearities and sensor-induced uncertainties.

HydroNeuro integrates these data-driven neural networks with classical hydraulic principles and a structured experimental design to form a practical and robust framework for real-time hydraulic network management. While the neural network model predicts pressures from observed data, the physical equations governing pressure dynamics (Equations (2) and (3)) provide a scientific basis for interpreting pressure deviations and validating the relevance of detected anomalies.

### 3.7. Embedded AI Pipeline with Edge Implementation

HydroNeuro combines IoT sensing and embedded AI for real-time, autonomous operation. Each step directly leverages the physical and statistical formulations described previously in Equations (2)–(7).

- **Data acquisition:** We measure nodal pressures $P_{r}$ across the network using sensors.For each valve configuration, measurements are not recorded instantaneously but over a stabilization window of 15 s. This delay allows transient hydraulic dynamics to dissipate and ensures that the system reaches a quasi-steady-state regime. The recorded value is then computed as a representative stabilized pressure, improving measurement reliability and reducing noise induced by fluid fluctuations and sensor latency.
- **FFD-based experimental selection and Gaussian augmentation:** We systematically vary valve states $V_{i}$ to explore main and interaction effects as formulated in the fractional factorial design (Equation (4)), leading to the following defining relation:
  $$I=V_{1}V_{2}V_{3}V_{4}.$$
  This Resolution IV design ensures that main effects are not aliased with other main effects or with two-factor interactions, while some interaction terms remain confounded (e.g., $V_{1}V_{2}\equiv V_{3}V_{4}$). The experimental design is further strengthened by repeated acquisitions, resulting in 13 total samples, thereby improving statistical robustness and enabling variance estimation.
  To enhance model generalization under noisy conditions, Gaussian data augmentation is applied:
  $${\tilde{P}}_{r}=P_{r}+N\left(0,\sigma^{2}\right)$$
  This augmentation simulates realistic perturbations and mitigates overfitting in small datasets.
- **Model training and optimization:** We first implement the MLR baseline (Equation (8)) to capture linear relationships between valve activations and pressures.
  The MLR model is expressed as follows:
  $$P_{r}=\beta_{0}+\sum_{i=1}^{4}\beta_{i}V_{i}+\epsilon$$
  and serves as a reference to evaluate the presence of nonlinear hydraulic interactions.
  We then train the MLP (Equation (10)) to model nonlinear dependencies and complex hydraulic interactions [27].
  The nonlinear mapping is learned using a multilayer perceptron:
  $${\hat{P}}_{r}=f\left(V_{1},V_{2},V_{3},V_{4};\;\theta \right)$$
  The network is trained for 1000 epochs using the Adam optimizer, enabling convergence and accurate modeling of higher-order hydraulic behaviors.
- **Conversion to TensorFlow Lite Micro:** The trained models are converted to lightweight embedded formats for deployment on microcontrollers.
  The conversion pipeline follows a multi-stage process: PyTorch 1.12.1
  (.pth) → ONNX → TensorFlow Lite (TFLite), followed by quantization and transformation into a C-compatible header file (.h). This pipeline significantly reduces memory footprint and inference latency while preserving predictive performance.
- **Deployment to ESP32 boards:** The edge devices perform real-time predictive inference, continuously estimating ${\hat{P}}_{r}$ based on current valve states.
  Because inference is executed locally on ESP32 microcontrollers, the system operates without dependency on cloud infrastructure [28]. This edge computing architecture minimizes latency, enhances robustness, and ensures reliable operation in environments with limited connectivity.
- **Continuous evaluation for anomaly detection:** The predicted pressures ${\hat{P}}_{r}$ are compared against measured values $P_{r}$ to detect deviations.
  The deviation is quantified as follows:
  $$\Delta P=|P_{r}-{\hat{P}}_{r}|$$
  A statistical decision threshold is defined as follows:
  ε=0.677753
  derived from the residual distribution using the three-sigma rule.
  If ΔP>ε, the system flags a potential anomaly, which may correspond to leakage, obstruction, valve malfunction, or sensor drift. This continuous monitoring framework enables early fault detection and supports predictive maintenance strategies.

Overall, the proposed embedded AI pipeline ensures an end-to-end integration from structured experimental design to real-time edge inference. By combining physically grounded data acquisition, statistically efficient experimental design, and lightweight embedded deployment, the system achieves a balance between accuracy, computational efficiency, and operational autonomy in hydraulic monitoring applications.

### 3.8. Embedded System Implementation and Operational Workflow

We design the HydroNeuro framework for fully offline operation, minimizing reliance on continuous Internet connectivity and ensuring practical applicability in rural and semi-arid agricultural regions.

We start with an initial dataset collection performed via a local Wi-Fi network over a period of 9 min, generating the necessary training data for the predictive model. Once trained, we convert the neural network model into a .h file and upload it to the ESP32 microcontroller through over-the-air (OTA) deployment via the same local Wi-Fi connection [17].

Once deployed, HydroNeuro operates in real time. We receive valve activation commands via LoRa communication, continuously acquire pressure measurements from embedded sensors, and perform predictive inference locally. We detect anomalies such as leaks or obstructions by comparing measured pressures with the reference predictions according to the thresholds defined in Equation (9). Anomalies are flagged immediately, enabling proactive and accurate monitoring across diverse hydraulic configurations [4,11].

HydroNeuro’s modular and scalable design accommodates a wide range of agricultural hydraulic systems, including conventional multi-sector irrigation, drip networks, and hydroponic setups. By combining IoT sensing, embedded artificial intelligence, and hybrid communication protocols, we deliver a sustainable, autonomous, and adaptable solution for intelligent hydraulic management in resource-constrained settings [4,9,14].

By systematically combining fractional factorial design with local pressure measurements, the HydroNeuro framework captures the most influential valve interactions while remaining computationally tractable for embedded deployment. This structured methodology naturally supports real-time anomaly detection and provides a foundation for evaluating predictive accuracy and operational performance across diverse irrigation scenarios.

To validate the framework prior to field implementation, we develop a physical prototype that serves as a pre-deployment experimental platform. This allows systematic experimentation under controlled conditions and ensures that observed pressure deviations can be meaningfully interpreted.

## 4. Experimental Setup and Practical Validation

The HydroNeuro prototype emulates an agricultural hydraulic network under controlled laboratory conditions. The scaled testbed, with pipe length L=1.25 m and diameter D=0.022 m, enables precise reproduction of valve configurations, pressure variations, leak scenarios, and obstruction events, providing a safe and resource-efficient environment for experimentation.

This setup allows direct comparison between predicted pressures ${\hat{P}}_{r}$ and measured pressures $P_{m}$, ensuring rigorous assessment of HydroNeuro’s predictive accuracy and operational reliability. A subsequent phase will focus on deployment and validation on a real agricultural installation under actual field conditions.

### 4.1. Experimental Design Refinement and Statistical Modeling

Building upon the fractional factorial framework introduced previously, the experimental design is further refined to improve the representativeness of the hydraulic operating space while maintaining a reduced experimental budget.

For the four-valve network (N=4), the baseline $2^{4-1}$ fractional factorial design provides 8 configurations capturing all main effects and selected two-factor interactions. However, this reduced design does not fully cover certain physically relevant operating regimes. To address this limitation, the experimental campaign is extended through targeted augmentation strategies.

First, four additional configurations corresponding to single-valve activation states (Hamming weight equal to 1) are introduced. These configurations enable direct estimation of individual valve contributions, improving parameter identifiability and reducing bias in main-effect estimation.

Second, a high-activation boundary condition (v=[1,1,1,0]) is added to capture nonlinear hydraulic behavior under near-saturation flow regimes. This addition mitigates extrapolation effects in subsequent learning models, effectively transforming the upper region of the operating space into an interpolation domain.

The resulting experimental dataset consists of n=13 configurations, as summarized in Table 1, providing a structured yet expressive sampling of the hydraulic response surface.

The pressure response model is defined as follows:

(11) $$P=\mu +\sum_{i=1}^{N}\beta_{i}V_{i}+\sum_{i<j}\beta_{ij}V_{i}V_{j}+\varepsilon$$

### 4.2. ANOVA-Based Factor Significance Analysis

The statistical structure of the model is evaluated using ANOVA, decomposing total variability into explained and residual components. The model explains a dominant proportion of the observed variance ($R^{2}=0.987$, $R_{\mathrm{adj}}^{2}=0.924$), confirming that the reduced experimental design captures the essential hydraulic dynamics.

All main effects are statistically significant (p<0.05), indicating that each valve induces a strong individual pressure drop. Valve V2 exhibits the highest contribution, highlighting its dominant influence.

Among interaction terms, only V2:V3 is significant at the α=0.05 level, revealing a non-additive hydraulic coupling consistent with flow redistribution in parallel branches.

### 4.3. Physical Interpretation and Modeling Implications

The estimated coefficients confirm physically consistent behavior: valve openings induce pressure decreases, while positive interaction terms reflect compensatory effects due to flow redistribution. These results align with classical hydraulic theory.

The presence of statistically significant interactions justifies the transition toward nonlinear modeling approaches (MLP), as linear models cannot fully capture these coupled dynamics under complex operating conditions.

### 4.4. Statistical Limitations

The limited number of residual degrees of freedom ($DF_{\mathrm{residual}}=2$) increases the sensitivity of statistical indicators. Therefore, results should be interpreted as identifying dominant structural effects rather than providing strict inferential conclusions.

### 4.5. Residual-Based Anomaly Detection Framework

Anomaly detection is performed using a residual-based approach. A neural model (MLP, architecture 4–32–32–1) is trained on normal operating conditions.

The anomaly score is defined as follows:

(12) $$\Delta P=\left|P_{r}-{\hat{P}}_{r}\right|$$

A detection threshold is derived using the three-sigma rule:

(13) $$\varepsilon =\mu_{\Delta P}+3\;\sigma_{\Delta P}$$

with $\mu_{\Delta P}=0.102977$ bar and $\sigma_{\Delta P}=0.191592$ bar, leading to $\varepsilon^{*}=0.677753$ bar.

**Figure 2:**
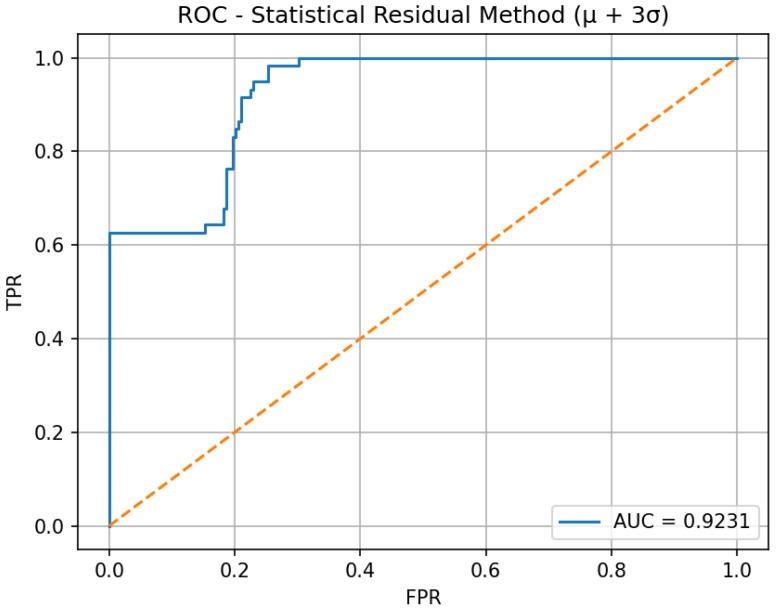
ROC curve for HydroNeuro anomaly detection. The selected threshold $\varepsilon^{*}=0.677753$ bar yields TPR =98.31% and FPR =25.36%. The Area Under the Curve (AUC) is 0.9231.

   Figure 2 shows the ROC curve for the anomaly detection model. ROC analysis evaluates detection performance, yielding an AUC of 0.9231, indicating strong discriminative capability. The selected operating point achieves a high detection rate with controlled false alarms.

### 4.6. Dataset Characteristics

The experimental campaign involved N=4 valves. We evaluated a total of 13 configurations (8 from the fractional factorial design, 4 additional configurations selected using Hamming weight criteria, and 1 empirically validated high-activation boundary state v=[1,1,1,0]). We measured each configuration three times, resulting in the following:

(14) $$N_{\mathrm{real}}=13\times 3=39\;\mathrm{physical}\;\mathrm{measurements}$$

Each physical measurement lasted 15 s, yielding a total experimental acquisition time of

(15) $$T_{\mathrm{total}}=39\times 15=585\;s=9.75\;\min\approx 0.163\;h$$

The limited acquisition duration highlights the efficiency of the proposed experimental design strategy. By reducing the number of required hydraulic configurations while preserving dominant interaction effects, the framework minimizes water usage, pump operating time, and mechanical stress on irrigation infrastructure during the data collection phase. This resource-aware methodology is especially relevant for sustainable agricultural systems, where experimental campaigns must avoid excessive water discharge and energy consumption.

We acquired pressure measurements from a single sensor in the hydraulic network along with the corresponding valve states (ON/OFF). For each configuration, we extracted the representative pressure value for learning from the stabilized response during the 15-s acquisition window.

#### 4.6.1. Dataset Splitting and Experimental Protocol

Prior to model training, the full dataset of $N_{\mathrm{total}}$ = 1339 samples was randomly shuffled to eliminate any unintended ordering bias that could arise from the chronological sequence of experimental acquisition. The shuffled dataset was then partitioned into a training set and a held-out test set according to an 80/20 ratio:

(16) $$N_{\mathrm{train}}=\left\lfloor 0.80\times 1339\right\rfloor =1071\;\mathrm{samples},\;N_{\mathrm{test}}=1339-1071=268\;\mathrm{samples}$$

This partitioning strategy is justified by three complementary considerations. First, allocating 80% of the samples to the training set provides both the MLR and the MLP with sufficient data to estimate the nonlinear mapping between valve states and nodal pressures—a requirement that is particularly critical for the MLP, whose generalization capacity depends on the diversity of the training distribution. Second, reserving 20% of the samples as a held-out test set ensures that final performance metrics are computed on strictly unseen data, providing an unbiased estimate of generalization performance that is independent of any training-time optimization. Third, the prior random shuffling step guarantees that both subsets contain representative samples drawn from all 13 hydraulic operating states, preventing the chronological clustering of specific valve configurations in either partition.

The 80/20 split represents a widely adopted standard in machine learning practice for datasets of moderate size, as it offers a principled compromise between maximizing training diversity and preserving a statistically meaningful evaluation subset. In the present context, it ensures that the held-out test set contains approximately 268/13≈20 samples per operational state on average, providing sufficient coverage to assess model performance across the full range of hydraulic configurations encountered during the experiment.

#### 4.6.2. Dataset Summary

Table 2 consolidates the key parameters of the dataset construction and augmentation pipeline for full reproducibility.

### 4.7. Threshold Sensitivity and Adaptive Detection Strategy

The threshold $\varepsilon^{*}=0.677753$ bar was derived from the residual distribution of the prototype system under controlled conditions using the statistical rule defined in Equation (13), with $\mu_{\Delta P}=0.102977$ bar and $\sigma_{\Delta P}=0.191592$ bar. Because this work is a proof of concept conducted on a stable 4-valve physical prototype, the experimental environment was relatively controlled, and a fixed threshold is appropriate for demonstrating the feasibility of edge-based anomaly detection. Under these conditions, the selected threshold yielded TPR =98.31% and FPR =25.36%.

However, in real irrigation or industrial hydraulic networks, several factors may progressively shift the nominal residual distribution: environmental noise, component aging, fluctuating inlet conditions, and pump degradation. A permanently fixed threshold would therefore progressively lose its calibration relevance, leading to either increased false alarms or reduced detection sensitivity over time.

For this reason, future large-scale deployments should rely on an **adaptive thresholding mechanism** rather than a fixed value of ε. A practically suitable strategy consists of recomputing the threshold from a sliding window of previously validated healthy states:

(17) $$\varepsilon_{t}=\mu_{\Delta P}^{\left(t\right)}+3\;\sigma_{\Delta P}^{\left(t\right)}$$

where $\mu_{\Delta P}^{\left(t\right)}$ and $\sigma_{\Delta P}^{\left(t\right)}$ denote the mean and standard deviation of residuals computed over a recent window of *W* validated normal observations ending at time *t*. This formulation allows the decision boundary to evolve continuously as the hydraulic system drifts, maintaining detection sensitivity while suppressing false alarms under dynamic operating conditions.

### 4.8. Testbed Architecture and Experimental Platform

**Figure 3:**
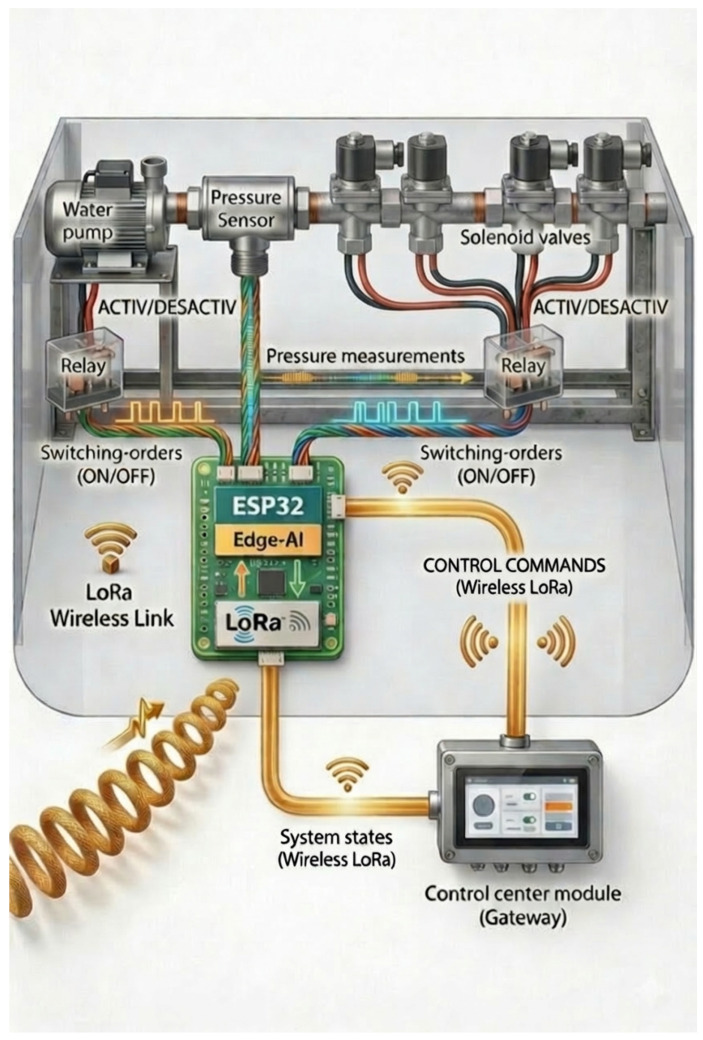
Experimental architecture of the HydroNeuro setup integrating sensors, actuators, and communication modules for real-time hydraulic monitoring and anomaly detection.

Figure 3 illustrates the experimental architecture designed for the HydroNeuro validation. The system includes a 24 V pump, solenoid valves, a pressure transducer (0–0.8 MPa range), and an ESP32-WROOM-32D microcontroller

(Espressif Systems, Shanghai, China) serving as the edge computing node.

We perform model training offline on a local PC using the data collected from the testbed. After training, the model is converted into a .h file and deployed to the ESP32 for real-time inference. During live operation, data are transmitted via LoRa for continuous anomaly reporting.

Such physical testbeds are crucial in the validation of IoT-based smart irrigation systems, as they allow the reproduction of hydraulic network behaviors in a manageable laboratory environment [2,7,17]. This approach ensures both scientific repeatability and the capacity to evaluate system responses under controlled perturbations.

### 4.9. Hydraulic Prototype and Component Integration

**Figure 4:**
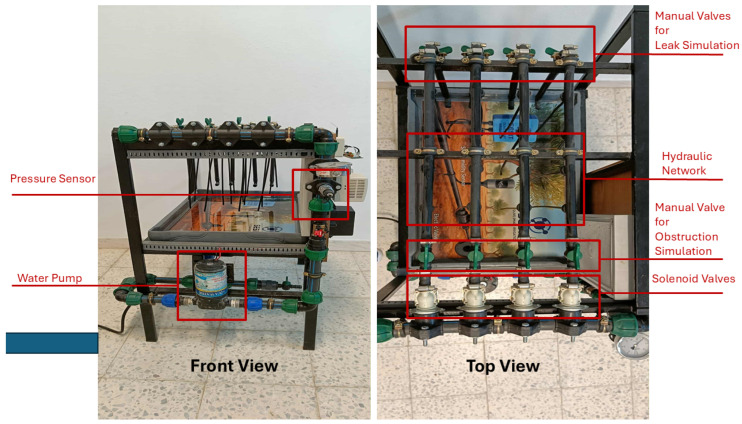
Physical prototype of the HydroNeuro system showing the hydraulic layout, sensors, and control valves.

We construct a dedicated hydraulic prototype (Figure 4) to replicate a small-scale irrigation network. The system includes 4 branches, 4 solenoid valves, and 1 pressure sensor, allowing us to test the data acquisition, inference, and anomaly detection modules under realistic operational conditions.

We design the prototype with a modular architecture, enabling flexible configuration of the number of valves, pressure sampling points, and flow paths. We use this setup to generate datasets under both normal and faulty scenarios, including simulated leaks and obstructions. Such experimental validation approaches have been widely adopted in precision agriculture studies leveraging IoT and embedded AI frameworks [3,8,24].

### 4.10. Custom PCB Design and System Integration

**Figure 5:**
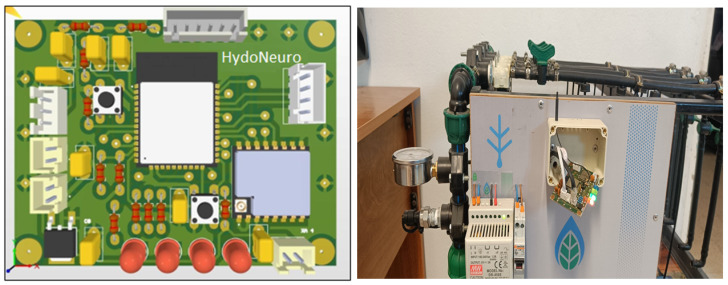
Custom PCB designed for the HydroNeuro system enabling compact and reliable hardware integration.

We design a custom PCB specifically for HydroNeuro (Figure 5) to ensure robust signal acquisition and low-noise operation. The PCB integrates:

- Command circuitry for the 24 V pump and solenoid valves.
- Analog signal conditioning for the pressure transducer.
- UART and Wi-Fi interfaces for real-time communication with the ESP32.

We leverage the ESP32 as the edge computing platform for on-device AI inference, taking advantage of its low cost, energy efficiency [29], and Wi-Fi/LoRa connectivity, which are well-suited for smart agriculture applications [15,16]. By using a dedicated PCB, we enhance electrical stability and reduce electromagnetic interference, ensuring reliable data acquisition and long-term system robustness [9].

### 4.11. Experimental Protocol and Validation

The experiments followed a structured protocol:

1. **Normal operation phase:** All valves were tested under standard flow conditions to establish baseline pressure references.
2. **Leak simulation:** One or multiple valves were partially opened to induce controlled pressure drops.
3. **Obstruction simulation:** Flow restrictions were introduced in selected branches to emulate partial blockages.

For each scenario, the predicted pressure from the deployed AI model (${\hat{P}}_{r}$) was compared with the measured pressure ($P_{m}$). Anomalies were detected when $|{\hat{P}}_{r}-P_{m}|>\epsilon$, where ϵ is determined using ROC-based optimization to maximize both sensitivity and specificity.

## 5. Results

To evaluate and optimize system performance, we acquire experimental data from the HydroNeuro testbed operating under controlled hydraulic conditions. We develop two predictive models—a conventional multiple linear regression (MLR) and a multilayer perceptron (MLP) neural network—as the primary comparative framework. To further substantiate the model selection process, we subsequently extend this analysis to a broader family of machine learning architectures, namely Decision Trees, Random Forests, and a lightweight 1D Convolutional Neural Network (1D-CNN), and systematically evaluate their feasibility for real-time embedded deployment on resource-constrained hardware.

This progressive, two-stage evaluation enables a systematic assessment of the framework’s precision, robustness, and ability to capture nonlinear hydraulic dynamics. By contrasting linear and nonlinear modeling performances, and by rigorously analyzing the embedded deployment constraints of each candidate architecture, we gain critical insights into HydroNeuro’s capacity for accurate and adaptive anomaly detection. Importantly, the objective of this extended analysis is not to provide an exhaustive algorithmic benchmark, but to identify the architecture that best satisfies the dual requirement of predictive accuracy and real-time deployability on the ESP32 microcontroller, the target execution platform of this work.

### 5.1. Linear Regression Model Evaluation

We develop the MLR model, as defined in Equation (8), to predict reference pressures across multiple hydraulic nodes. The predicted pressures ${\hat{P}}_{r}$ are compared against the measured pressures $P_{m}$, which serve as ground truth acquired directly from the pressure sensors embedded in the HydroNeuro prototype.

The model is evaluated under a range of hydraulic configurations, encompassing different valve activation patterns and flow paths as illustrated in the second subfigure of Figure 5. These conditions are specifically designed to capture both nominal operation and scenarios involving localized hydraulic disturbances, providing a representative test of model robustness.

A critical observation is that the linear regression model occasionally predicts negative pressures, which are physically inadmissible. These anomalous predictions stem from the absence of physical constraints in the model formulation and from the limited expressive capacity of linear mappings when confronted with nonlinear hydraulic behaviors, multi-valve interactions, and localized head losses inherent to the network topology.

Although the model partially captures the global trend of pressure variations, it exhibits clear *high-bias (underfitting)* behavior. This systematic bias is not reducible to a simple constant offset; consequently, post hoc affine corrections are insufficient to recover accurate predictions under complex or faulty hydraulic configurations.

Quantitatively, the MLR model achieves a root mean squared error (RMSE) of 0.58 bar, corresponding to an MSE of approximately 0.34 bar^2^. Given an operating pressure range of approximately 0.25 MPa, this error magnitude represents a non-negligible relative deviation that is incompatible with the precision requirements of high-fidelity hydraulic anomaly detection.

These results collectively establish the intrinsic limitations of linear regression for this class of problems and provide strong motivation for adopting nonlinear learning architectures capable of representing the complex pressure–flow dynamics of agricultural hydraulic networks.

**Figure 6:**
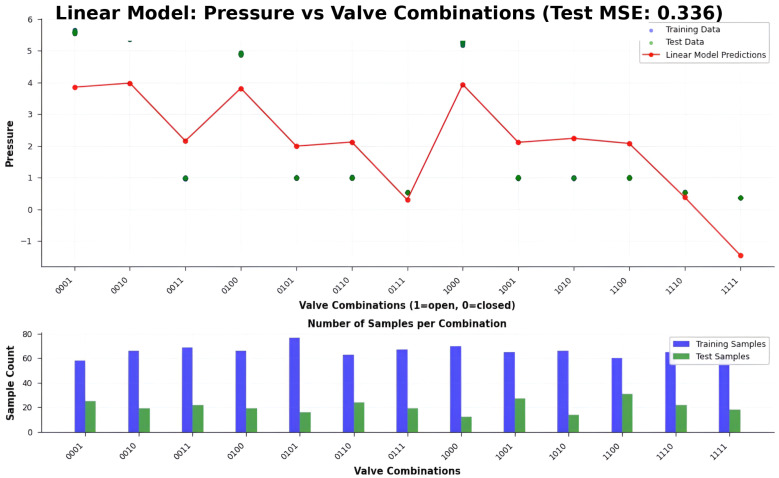
Performance of the linear regression model in pressure prediction across valve combinations (Test MSE = (0.336)).

Figure 6 illustrates the limitations of the linear regression model in predicting hydraulic pressures under varying valve configurations. The model occasionally produces physically invalid negative pressures and exhibits significant deviations from the measured ground truth ($P_{m}$). These observations highlight the inability of linear models to capture complex nonlinear interactions in the network, motivating the use of nonlinear approaches such as neural networks for accurate anomaly detection.

### 5.2. Neural Network Model Evaluation

We develop a feedforward multilayer perceptron (MLP) and deploy it on the ESP32 platform to model the nonlinear relationships between valve configurations and nodal pressure responses in the hydraulic network. The final architecture was selected through iterative empirical validation while remaining explicitly constrained by the memory and computational budget of the target embedded platform.

#### 5.2.1. Structural Configuration and Hardware Constraints

The adopted architecture consists of an input layer of dimension nb_valves where nb_valves denotes the number of controllable valves in the hydraulic configuration followed by two fully connected hidden layers of 32 neurons each, and a single linear output neuron for continuous pressure regression. The full model pipeline is defined as follows:

(18) $$\begin{array}{cc} \underset{\mathrm{hidden}\;\mathrm{layer}\;1}{\underset{︸}{\mathrm{Linear}(\mathrm{nb}\text{\_}\mathrm{valves},\;32)}} & \to \mathrm{ReLU}\to \mathrm{Dropout}(0.1)\\ \; & \to \underset{\mathrm{hidden}\;\mathrm{layer}\;2}{\underset{︸}{\mathrm{Linear}(32,\;32)}}\to \mathrm{ReLU}\\ \; & \to \underset{\mathrm{output}}{\underset{︸}{\mathrm{Linear}(32,\;1)}} \end{array}$$

This configuration provides sufficient representational capacity for the nonlinear valve-state-to-pressure mapping while satisfying the hardware constraints of the ESP32. Deeper architectures with three or more hidden layers, or significantly wider layers, were evaluated and systematically discarded: they increase RAM usage and inference latency without yielding measurable accuracy gains on this low-dimensional regression task. The compact two-hidden-layer topology of 32 neurons per layer therefore constitutes an intentional and evidence-based compromise between nonlinear expressive power and embedded deployability.

#### 5.2.2. Training Protocol and Generalization Assessment

The model is trained offline on a workstation for 1000 epochs with a batch size of 32, using a training procedure designed to ensure both convergence stability and generalization robustness. Training and testing partitions are strictly separated prior to any fitting step to eliminate data leakage. A *k*-fold cross-validation procedure is applied to assess generalization stability, yielding an RMSE standard deviation below 5% across folds, confirming consistent performance across data partitions.

#### 5.2.3. Activation, Optimization, and Regularization Strategy

Each hidden layer uses the Rectified Linear Unit (ReLU) activation function, selected for its computational efficiency, straightforward implementation on embedded hardware, and proven effectiveness in learning nonlinear mappings without introducing vanishing gradient effects. A linear activation is applied at the output layer to preserve the continuous range of predicted pressure values.

The network is optimized using the Adam optimizer with an initial learning rate of $\eta =10^{-2}$, selected for its adaptive step-size behavior and robust convergence in nonlinear regression tasks. The MSE loss function is used as the training objective, providing a differentiable and physically meaningful measure of prediction error in pressure units. To mitigate overfitting and improve parameter stability, L2 regularization with a weight decay of $\lambda =1\times 10^{-5}$ is applied globally during optimization.

Three complementary regularization mechanisms are incorporated into the training procedure to ensure robust generalization:

- **Dropout regularization:** A dropout layer with probability p=0.1 is inserted after the first hidden layer. This stochastic mechanism discourages co-adaptation of neurons during training and promotes the learning of more distributed and stable feature representations of the hydraulic input space.
- **Adaptive learning rate scheduling:** A ReduceLROnPlateau scheduler is employed with a patience of five epochs and a halving factor of 0.5. Whenever the validation loss plateaus, the learning rate is automatically reduced, enabling finer convergence toward flat loss minima and suppressing oscillations in the late training phase.
- **Validation-based convergence control:** Although no explicit early-stopping callback is enforced, the validation loss trajectory is monitored continuously throughout training. The adaptive scheduling strategy ensures that effective learning terminates naturally once generalization performance ceases to improve, preventing unnecessary overfitting to the training partition.

Collectively, these design choices confirm that the proposed MLP architecture is not empirically arbitrary but results from a principled optimization of the trade-off between nonlinear predictive capacity, regularization robustness, and hardware-aware deployability on the ESP32 microcontroller.

#### 5.2.4. Embedded Conversion and Deployment

Following training, the model is converted to **TensorFlow Lite Micro (TFLM)** format and quantized using an 8-bit scheme to reduce memory footprint and accelerate on-device inference. The deployed model occupies less than 20 kB of RAM during execution on the ESP32, satisfying the stringent memory constraints of low-resource edge platforms while fully preserving predictive accuracy.

#### 5.2.5. Predictive Performance

**Figure 7:**
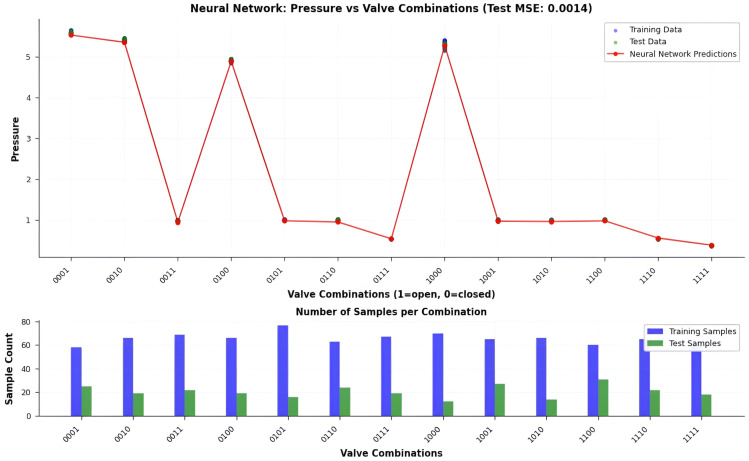
Neural network pressure prediction versus valve combinations (training and test results). The predicted pressures ${\hat{P}}_{r}$ closely track the measured ground truth $P_{m}$ across all tested hydraulic configurations, confirming the ability of the MLP to generalize across diverse valve activation patterns.

The prediction results (Figure 7) demonstrate a significantly closer alignment between ${\hat{P}}_{r}$ and $P_{m}$ compared to the linear baseline. The MLP achieves an RMSE of 0.12 bar, representing an order-of-magnitude improvement over the MLR. These results confirm that the neural network effectively captures nonlinear flow dynamics, valve–valve interaction effects, and localized hydraulic disturbances that are structurally inaccessible to linear models. Despite its black-box nature, the model delivers robust and accurate pressure estimation, establishing its suitability for high-fidelity anomaly detection in complex agricultural hydraulic networks.

### 5.3. Comparative Accuracy Analysis

**Figure 8:**
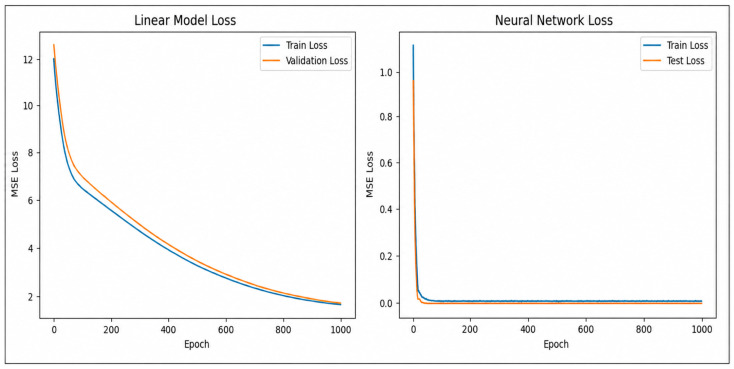
Comparison of training and testing MSE curves for linear regression and neural network models. The MLR model is trained using gradient-based optimization and converges rapidly toward a plateau at MSE≈0.34, reflecting its limited expressive capacity. In contrast, the MLP exhibits a continuous reduction in loss over 1000 epochs, achieving MSE<0.02, demonstrating its ability to model complex nonlinear hydraulic relationships.

**Figure 9:**
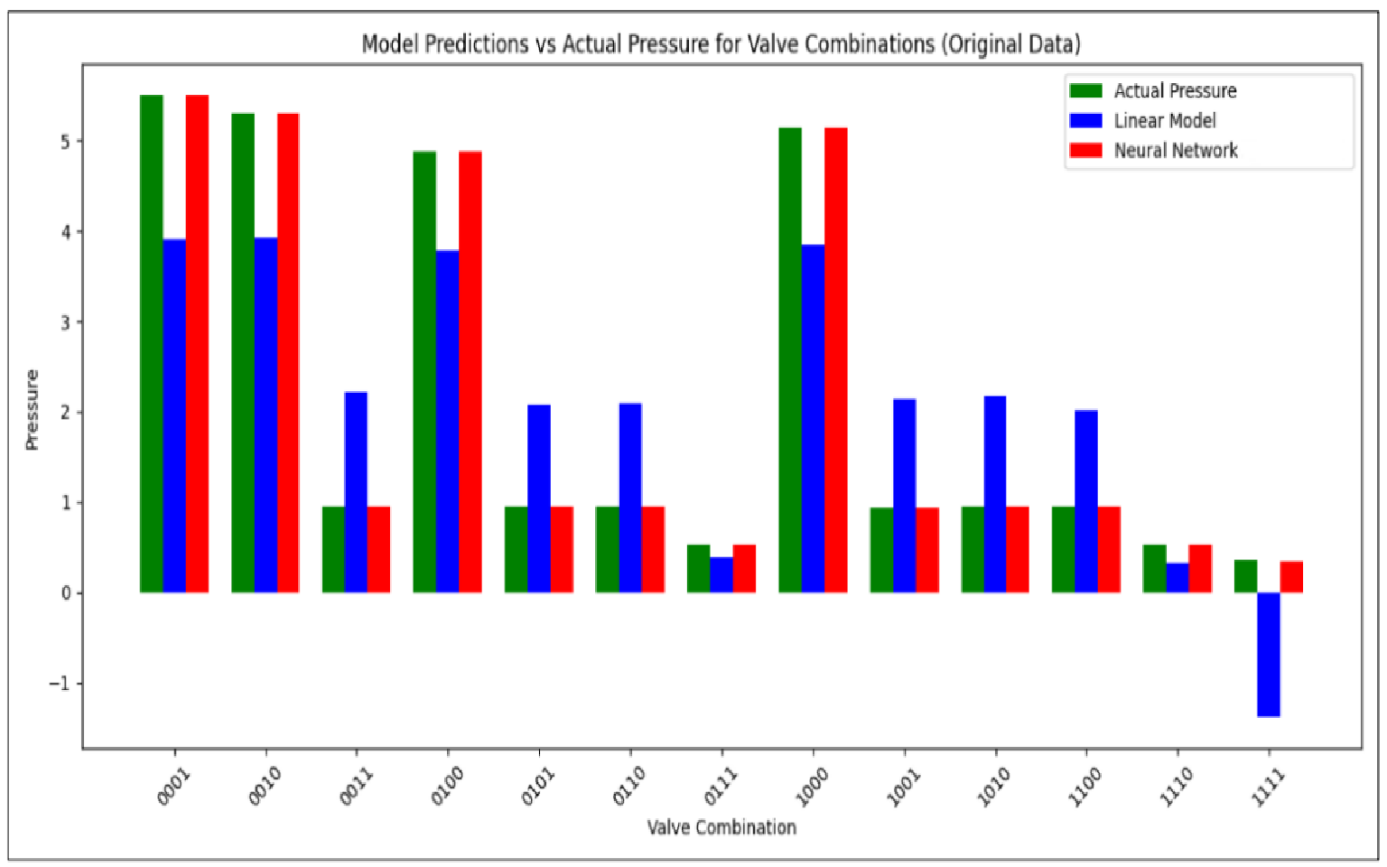
Bar chart comparison of prediction performance for the two models.

A direct comparison between the two models confirms the superiority of the neural approach. Figure 8 displays the comparative accuracy curves, while Figure 9 summarizes predictive performance using a bar plot.

We observe that the MLR baseline achieves a mean squared error (MSE) of approximately 0.34 after convergence. Although linear regression admits a closed-form Ordinary Least Squares (OLS) solution, in this work the MLR model is trained using a gradient-based optimization procedure over multiple epochs. This implementation choice ensures methodological consistency with the MLP training process and enables a direct comparison of learning dynamics between the two models. As shown in Figure 8, the MLR loss decreases rapidly during the initial training phase and then stabilizes at a plateau around MSE≈0.34. This convergence behavior reflects the limited expressive capacity of linear models: while optimization reduces the training error, the model quickly reaches its bias-limited performance and cannot further improve due to its inability to capture nonlinear relationships.

This residual error reflects the inherent limitation of linear regression in modeling the nonlinear pressure–flow relationships arising from multi-valve interactions, frictional head losses, and local turbulence phenomena. The presence of a non-negligible error plateau indicates a dominant bias component, confirming that linear parametrizations lack the expressive capacity required to accurately represent the hydraulic dynamics of the system.

In contrast, we train the MLP over 1000 epochs with a batch size of 32. The extended training horizon improves convergence stability and allows the model to progressively capture higher-order nonlinear interactions. The network rapidly reduces both training and validation MSE, converging to MSE<0.02 well before the final epoch. The close alignment of the training and validation curves throughout the full training horizon demonstrates effective generalization. This stability results from the combination of regularization strategies and adaptive learning rate behavior, which collectively prevent overfitting despite the relatively limited number of experimental samples.

We find that the MLP’s superior expressive power allows it to capture subtle nonlinear hydraulic phenomena, including compounded head losses, valve–valve interaction effects, partial-flow recirculation, and local venturi-like pressure reductions. These dynamics, governed by the interplay between Bernoulli-driven pressure variations and dissipative mechanisms described by the Darcy–Weisbach framework, are intrinsically inaccessible to linear models.

The loss curves in Figure 8 clearly illustrate this contrast: while the MLR exhibits a rapid convergence followed by a saturation plateau, the MLP demonstrates a continuous decrease in error, reaching significantly lower MSE values. This behavior confirms the necessity of nonlinear architectures for accurate hydraulic state prediction and validates the suitability of the MLP for embedded anomaly detection in HydroNeuro.

The adopted MLP architecture consists of two hidden layers, each containing 32 neurons, forming a compact yet expressive topology suitable for embedded deployment. We select this configuration to balance nonlinear modeling capability with the memory and computational constraints of the ESP32 microcontroller. We use Rectified Linear Unit (ReLU) activation functions in the hidden layers to introduce nonlinearity and mitigate vanishing gradient effects, while applying a linear activation function at the output layer to predict continuous pressure values.

We train the network using the mean squared error (MSE) loss function, defined as the average squared difference between predicted pressures ${\hat{P}}_{r}$ and measured pressures $P_{m}$. The model is optimized over 1000 epochs using backpropagation with the Adam optimizer, selected for its adaptive learning rate mechanism and robust convergence properties in nonlinear regression tasks.

### 5.4. Comparative Performance Analysis

Table 3 consolidates the predictive performance of both models on the HydroNeuro testbed across four standard regression metrics.

The performance gap between the two approaches is substantial and consistent across all metrics. The MLR baseline exhibits high prediction errors (RMSE = 0.58 bar, MAE = 0.41 bar, $R^{2}=0.787$), which are directly attributable to its inability to represent nonlinear hydraulic interactions and valve coupling effects. The proposed MLP achieves RMSE = 0.12 bar and MAE = 0.08 bar, with a near-perfect coefficient of determination of $R^{2}=0.999$. This corresponds to an approximate **80% reduction in RMSE** relative to the linear baseline, confirming near-perfect agreement between predicted and measured pressures and establishing the MLP as a high-fidelity estimator of hydraulic state under realistic operational conditions.

### 5.5. Extended Model Comparison and Embedded Deployment Analysis

The two-model comparison presented in Section 5.1, Section 5.2, Section 5.3 and Section 5.4 conclusively establishes two findings: (i) the MLR is inadequate for this class of nonlinear hydraulic regression problems, and (ii) the MLP achieves near-perfect predictive accuracy within the embedded memory budget of the ESP32. The present section conducts a second, system-level evaluation stage to determine whether the MLP is not merely the most accurate candidate but also the most architecturally coherent choice for real-time firmware deployment among a broader family of machine learning models. This extended analysis is explicitly not intended as an exhaustive algorithmic benchmark; rather, it is guided by three system-level selection criteria: (i) deployment feasibility on resource-constrained hardware with limited flash, RAM, and computational throughput; (ii) deterministic, low-latency inference compatible with real-time hydraulic monitoring requirements; and (iii) sufficient nonlinear expressive power to represent the pressure–valve dynamics of the HydroNeuro network.

#### 5.5.1. Rationale for the Extended Model Family

The linear regression model, whose limitations are fully characterized in Section 5.1, and the MLP, whose architecture and deployment properties are detailed in Section 5.2, are not re-analyzed here. Their performance figures are reproduced in Table 4 and Table 5 solely as reference anchors for unified cross-model comparison. The following additional architectures are introduced specifically to contextualize the MLP’s position within a broader model family and to provide a rigorous, evidence-based justification for the architectural choices made in this work:

- **Decision Trees and Random Forests:** These methods are well established for tabular regression and achieve competitive predictive accuracy across many application domains. However, their embedded firmware representation is structurally incompatible with the deployment pipeline adopted in this work. Upon conversion via tools such as emlearn, tree-based models are expanded into branching-heavy executable C functions with high cyclomatic complexity. Unlike the compact passive data array produced by the TFLM pipeline, this logic-dense representation entangles model parameters with inference control flow, complicates firmware maintenance, and produces data-dependent execution paths that are difficult to predict on microcontrollers with limited instruction-cache capacity.
- **1D Convolutional Neural Networks (1D-CNN):** Although 1D-CNNs are effective for structured sequential feature extraction from time-series or spatial signals, their architectural inductive bias is not well matched to the compact, low-dimensional, non-sequential tabular input space of this application. In the present configuration, the input vector encodes discrete valve states and provides no structural advantage for convolutional processing. As a result, 1D-CNNs introduce additional computational overhead and parameter count without delivering expressive gains over the compact MLP.
- **Isolation Forests and Autoencoders:** These unsupervised architectures are designed for distribution-based anomaly scoring in the absence of labeled data. They are not applicable to the supervised continuous pressure regression task formulated in this work and do not constitute methodologically comparable baselines. They are therefore excluded from the quantitative evaluation.

#### 5.5.2. Quantitative Results for the Additional Architectures

Table 4 presents the predictive performance of the three additional architectures—Decision Tree, Random Forest, and 1D-CNN—evaluated on the same held-out test set (267 samples, 20% stratified split) used in Section 5.4. In addition to standard regression metrics, a composite Custom Score is reported, defined as follows:

(19) $$\mathrm{Custom}\;\mathrm{Score}=1-\frac{\mathrm{RMSE}}{\max_{M}\left(\mathrm{RMSE}\right)}$$

where the maximum is taken over all models in the extended candidate set M, including the MLR and MLP reported in Table 3. This normalized, bounded score quantifies inference quality relative to the worst-performing architecture, enabling direct cross-model comparison on a common scale.

All three additional nonlinear architectures achieve high predictive accuracy, consistent in order of magnitude with the MLP results reported in Table 3. The Decision Tree and Random Forest reach nearly identical performance to the MLP. A permutation test (10,000 iterations) between the MLP and the Random Forest yields p=0.3281, well above the conventional significance threshold of α=0.05, confirming that their performance difference is not statistically significant. The 1D-CNN achieves a marginally higher RMSE of 0.022 bar without any compensating architectural advantage for this low-dimensional, non-sequential input space. Under conditions of statistically equivalent predictive accuracy, architectural model selection is therefore governed exclusively by embedded deployment feasibility, which is analyzed in the following subsection.

#### 5.5.3. Embedded Firmware Representation Analysis

To provide a concrete, reproducible, and technically grounded assessment of deployment coherence, we analyze the structure of the firmware artifact generated by each model after conversion to an embedded-compatible format. This analysis is central to the architectural justification of the proposed approach.

#### 5.5.4. Neural Network (MLP) Compact Passive Data Representation

As detailed in Section 5.2, the trained MLP is serialized via the TFLM pipeline into a .tflite binary, subsequently converted into a C-compatible .h header file. The resulting firmware artifact consists of a single contiguous, memory-aligned hexadecimal byte array:


#include "model_data.h"
#include <cstdint>



alignas(8) const uint8_t converted_model_tflite[] = {
  0x1c, 0x00, 0x00, 0x00, 0x54, 0x46, 0x4c, 0x33,
  0x14, 0x00, 0x20, 0x00, 0x1c, 0x00, 0x18, 0x00,
  0x44, 0x49, 0x00, 0x00, 0x54, 0x49, 0x00, 0x00,
  0x43, 0x4f, 0x4e, 0x56, 0x45, 0x52, 0x53, 0x49,
  \dots
};


In this representation, the model is stored as passive, read-only data entirely decoupled from the inference control flow. All computation is delegated to a fixed, pre-compiled TFLM runtime engine operating on a statically pre-allocated memory arena. This architecture provides three critical embedded deployment properties: (i) **memory isolation**—the AI inference module cannot interfere with other concurrent real-time tasks executing on the ESP32; (ii) **deterministic RAM usage**—the arena size is resolved at compile time, eliminating dynamic allocation and ensuring predictable resource consumption; and (iii) **frictionless model updates**—redeploying a retrained model requires only replacing the hexadecimal array, with no modification to firmware logic or recompilation of the runtime.

#### 5.5.5. Decision Tree Branching Executable Logic

By contrast, a converted Decision Tree does not produce a passive data artifact but generates explicit branching prediction logic directly embedded in the firmware executable. A representative excerpt of the generated C artifact is shown below:


static inline float decision_tree_tree_0(
    const float32 *features, int32_t features_length) {
  if (features[1] < 0.5) {
    if (features[2] < 0.5) {
      if (features[0] < 0.5) {
        return 5.582143f;
      } else {
        if (features[3] < 0.5) {
          return 5.293289f;
        } else {
          return 0.986048f;
        }
      }
    }
  }
}


This structure violates the data logic separation principle that underpins the TFLM deployment pipeline. The model parameters are not stored as passive data but are encoded as executable branching conditions, which increases cyclomatic complexity, introduces data-dependent execution paths, exerts pressure on the microcontroller instruction cache, and significantly increases firmware maintenance cost: any model update requires regenerating the full function body and recompiling the firmware.

#### 5.5.6. Random Forest Massively Replicated Branching Structure

The firmware overhead is substantially amplified for Random Forest ensembles. After conversion, the full ensemble expands into a large family of individually generated tree functions, spanning from random_forest_tree_0(…) to random_forest_tree_99(…), each implementing its own nested if–else branching logic. A representative generated function is shown below:


static inline float random_forest_tree_0(
    const float32 *features, int32_t features_length) {
  if (features[1] < 0.5) {
    if (features[0] < 0.5) {
      if (features[2] < 0.5) {
        return 5.584759f;
      } else {
        if (features[3] < 0.5) {
          return 5.405064f;
        } else {
          return 0.977162f;
        }
      }
    }
  }
}


A fully converted Random Forest with 100 estimators produces 100 such functions, and the final prediction is obtained by averaging their individual outputs. The cumulative firmware artifact is therefore not a compact data representation but a large body of replicated branching logic that is instruction-cache-intensive, difficult to maintain, and incompatible with the clean separation between model data and inference runtime that characterizes the TFLM integration pattern. This structural overhead is entirely independent of predictive accuracy and represents a hard firmware engineering constraint in resource-limited real-time embedded systems.

#### 5.5.7. Unified Accuracy–Deployability Trade-Off

Table 5 synthesizes, for the first time across all five candidate architectures, the quantitative accuracy results from Table 3 and Table 4 together with the firmware representation analysis, providing a unified evaluation along both the predictive and deployment dimensions simultaneously.

The unified evaluation reveals a clear and unambiguous result. The MLP is the only architecture that simultaneously satisfies both the accuracy and deployment requirements of the HydroNeuro system. Tree-based models—Decision Tree and Random Forest—achieve predictive performance statistically equivalent to the MLP but fail the firmware coherence criterion due to their logic-dense, branching-heavy embedded representations. The 1D-CNN offers no accuracy advantage over the MLP while introducing additional computational overhead on a resource-constrained platform. The linear model, despite being natively deployable without conversion overhead, is excluded from deployment consideration on accuracy grounds, as fully established in Section 5.1.

This outcome directly and quantitatively supports the central architectural claim of this manuscript: the primary contribution of HydroNeuro lies in the accuracy–efficiency trade-off achieved by a lightweight deployable neural model operating under real-time firmware constraints, rather than in a purely algorithmic comparison across heterogeneous model families.

### 5.6. Summary of Results

The results presented in this section establish a coherent, two-stage and converging scientific conclusion. In the first stage (Section 5.1, Section 5.2, Section 5.3 and Section 5.4), the systematic comparison between the MLR and the MLP demonstrates that nonlinear modeling is strictly necessary for accurate hydraulic pressure estimation: the MLP delivers an approximate **80% reduction in RMSE** relative to the linear baseline (from 0.58 bar to 0.12 bar), with a coefficient of determination of $R^{2}=0.999$, while satisfying the embedded memory constraints of the ESP32 platform. In the second stage (Section 5.5), the extended model comparison confirms that, among all five evaluated architectures, the MLP uniquely combines statistically optimal predictive accuracy with full architectural coherence with the TFLM firmware deployment pipeline. The proposed neural architecture trained offline, converted into a compact hexadecimal passive data representation, and executed by an optimized runtime with statically pre-allocated memory and deterministic execution behavior constitutes the most rigorous and practically suitable solution for real-time hydraulic anomaly detection under the operational and hardware constraints of the HydroNeuro system.

### 5.7. Operational Validation in Practical Conditions

We validate HydroNeuro under realistic operating conditions using an Arduino controller with LoRa communication to send valve activation commands and receive anomaly alerts in real time. The embedded ESP32 executes the quantized MLP for each new pressure measurement.

**Figure 10:**
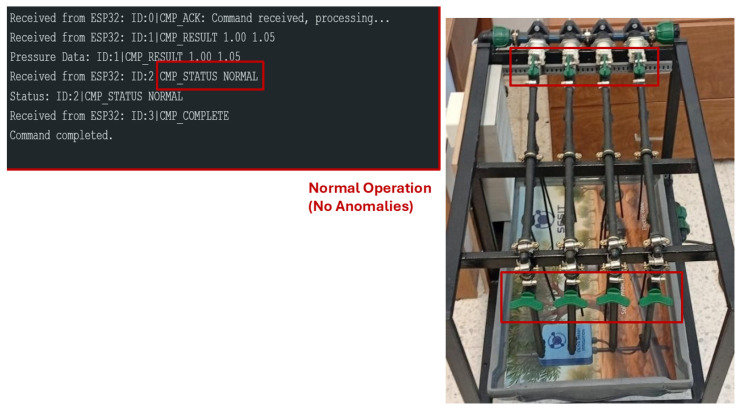
System performance under normal operating conditions.

During normal operation, the system correctly identifies the absence of anomalies, as shown in Figure 10. The predicted and measured pressures remain within the ROC-optimized threshold ϵ, confirming stable network behavior.

During normal operation, the hydraulic network functions without induced faults: all manually installed valves for leak simulation are closed, preventing any unintended water loss, while valves intended for obstruction testing remain open, allowing unrestricted flow. This configuration ensures that the measured pressures reflect standard network behavior, providing a baseline for anomaly detection using HydroNeuro.

**Figure 11:**
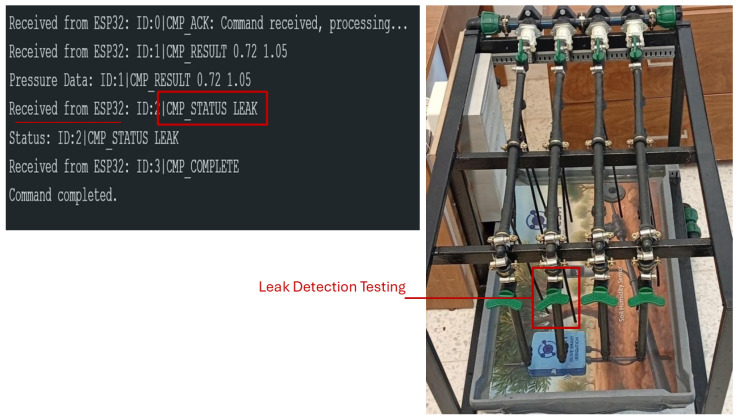
Leak simulation and corresponding anomaly detection response.

In a simulated leakage scenario (Figure 11), the system detects the characteristic pressure drop and immediately transmits a leak alert through LoRa communication. The embedded inference ensures a detection latency of approximately 5–10 s per measurement, enabling near-real-time response to hydraulic anomalies.

In the simulated leakage scenario, we deliberately open the valve installed for leak induction to create a controlled pressure drop, while all other valves, including those designated for obstruction simulation, remain closed. This setup ensures that the observed pressure changes are solely due to the induced leak, allowing HydroNeuro to accurately detect the anomaly.

**Figure 12:**
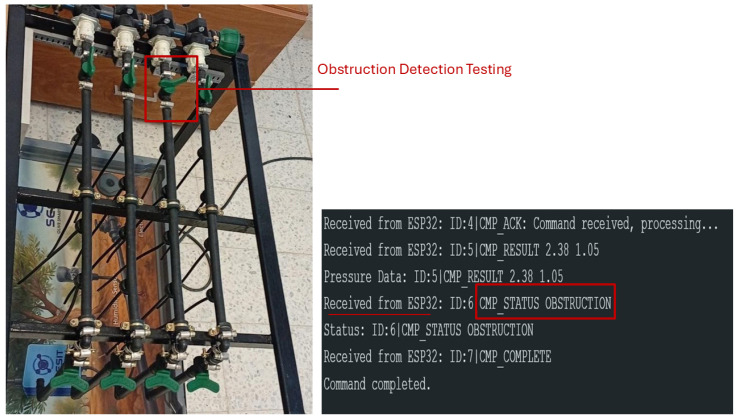
Obstruction simulation and anomaly detection.

In the obstruction scenario (Figure 12), the system accurately detected increased pressure due to the restriction and correctly classified the anomaly as an obstruction.

In the simulated obstruction scenario, we deliberately close the valve installed for obstruction induction to create a localized flow restriction, while all valves, those designated for leak simulation, remain closed. This configuration ensures that the observed pressure changes are caused solely by the induced obstruction, allowing HydroNeuro to accurately detect and respond to the anomaly in real time.

Across all experimental conditions, as summarized in Table 6, HydroNeuro achieves a detection accuracy exceeding **96%**, confirming the effectiveness of the hybrid physical–digital framework. These results validate HydroNeuro’s real-world performance and demonstrate reliable, real-time operation under realistic irrigation conditions, highlighting its capacity to support responsive and autonomous hydraulic monitoring in resource-constrained agricultural environments.

### 5.8. Detection Performance Discussion

In the proposed HydroNeuro framework, fault detection and localization are designed as a two-stage process combining global anomaly detection and sequential diagnostic refinement.

During normal operation, the system continuously monitors hydraulic behavior under arbitrary valve combinations. When a deviation from the learned pressure distribution is detected (indicative of leakage or obstruction), the system does not immediately attempt direct multi-fault classification. Instead, it initiates a structured diagnostic procedure restricted to the subset of actively involved branches within the detected configuration.

More specifically, once an anomaly is identified, the system performs a **sequential sector-wise activation strategy**, where each active branch is individually actuated while monitoring the corresponding pressure response. This controlled excitation process enables the system to isolate the faulty branch (or branches) by analyzing differential pressure deviations relative to expected nominal behavior.

Formally, this procedure can be interpreted as a localized sensitivity analysis, where each valve state $V_{i}$ is independently perturbed and the resulting pressure response $P_{i}$ is evaluated. The faulty branch is then identified as the one producing the most significant deviation from the learned pressure model. Importantly, this mechanism naturally extends to **multi-branch fault scenarios**, including multiple simultaneous leakages or multiple obstructions, as each branch is evaluated independently during the diagnostic phase.

Therefore, although the experimental validation presented in this work focuses on single-fault scenarios (single leakage or single obstruction), the proposed diagnostic architecture is inherently capable of isolating **multiple faulty branches** through sequential interrogation without requiring explicit multi-label training.

However, a specific limitation arises in the case of **simultaneous mixed faults**, where leakage and obstruction occur concurrently in different branches. From a hydraulic perspective, leakage typically induces a pressure drop, while obstruction may produce localized pressure increases or flow redistribution. In rare situations, these opposing effects may partially compensate for each other, resulting in **near-equilibrium pressure conditions** at the observation point.

In such cases, the global anomaly detection stage may fail to trigger because the combined pressure signature does not sufficiently deviate from the nominal distribution. This represents a **low-probability but theoretically possible blind spot** inherent to single-point pressure monitoring systems.

It is important to emphasize that this limitation corresponds to a highly specific and uncommon operating condition, requiring a precise balance between opposing hydraulic effects. As such, it does not affect the general validity of the proposed approach nor its effectiveness in practical irrigation scenarios, where faults typically produce non-compensating pressure deviations.

Addressing this edge case would require enhanced system observability, such as multi-point pressure sensing, flow measurements, or more advanced spatio-temporal modeling techniques capable of disentangling overlapping hydraulic signatures.

Overall, the proposed methodology provides a robust and scalable solution for both single- and multi-branch fault localization under realistic operating conditions, while clearly identifying the boundary conditions under which its performance may be limited.

### 5.9. Scalability Analysis for Larger Valve Networks

#### 5.9.1. Dataset Construction and Experimental Reduction

To evaluate scalability beyond the physical 4-valve prototype, a 10-valve configuration was analyzed using a simulation-based dataset generated in Python 3.8.15.

This approach enables controlled exploration of larger combinatorial spaces while maintaining consistency with the experimental design methodology adopted in the physical setup.

More generally, a two-level fractional factorial design is denoted as $2^{k-p}$, where *k* represents the total number of factors (valves) and *p* is the fraction parameter controlling the level of experimental reduction. The resulting number of experimental runs is therefore $2^{k-p}$. Increasing *p* reduces the number of required runs exponentially, but introduces controlled aliasing among higher-order interaction terms.

In practice, p=0 corresponds to a full factorial design, while larger values of *p* are used to enable efficient screening under constrained experimental budgets. A commonly accepted guideline is to maintain k−p≥3, ensuring a minimum of eight base runs and preserving statistical interpretability. For small-scale systems (k=4 to 7), *p* typically ranges between 1 and 3, whereas for medium-scale systems (k=8 to 12), values between 4 and 6 are commonly adopted to balance resolution and experimental cost.

In the context of this study, the physical four-valve system naturally corresponds to a half-fraction design $2^{4-1}=8$. For the 10-valve scalability analysis, we adopt a $2^{10-5}=32$ design, which represents a well-established compromise between coverage and efficiency. A full factorial exploration would require $2^{10}=1024$ configurations; thus, the adopted fractional design achieves a reduction of approximately 96.9% while preserving the main effects and the most relevant low-order interactions. This configuration is consistent with a Resolution IV design, ensuring that main effects remain unconfounded by other main effects, while accepting controlled aliasing among interaction terms.

For clarity, Table 7 summarizes typical values of *p* for different system sizes in two-level fractional factorial designs.

#### 5.9.2. Memory Footprint

To assess deployment scalability, the memory footprint of the trained models was evaluated after conversion to an embedded-compatible format. The 4-valve model occupies 4.06 KB, while the 10-valve model requires 4.44 KB.

This represents an increase of only 8.53%, demonstrating that the model size scales sub-linearly with respect to the number of input variables. Importantly, this footprint remains well within the memory constraints of the ESP32 platform, leaving sufficient resources available for concurrent sensing, communication, and control operations.

#### 5.9.3. Inference Latency

From a computational perspective, the complexity of the MLP grows approximately linearly with the input dimensionality, particularly in the first dense layer. Expanding the input from 4 to 10 valves increases the number of multiply–accumulate operations, but does not introduce exponential growth.

Given a hidden layer width of 32 neurons, the additional computational cost remains modest and well within the processing capabilities of the ESP32. Consequently, the inference latency remains compatible with real-time constraints required for hydraulic monitoring and anomaly detection.

#### 5.9.4. Engineering Interpretation

These results indicate that the proposed architecture maintains a favorable scaling behavior as the network size increases. The combination of compact model representation and near-linear computational growth ensures that the system can be extended to larger hydraulic infrastructures without violating embedded constraints.

Furthermore, the use of fractional factorial design enables efficient dataset construction even for high-dimensional systems, ensuring that the dominant hydraulic interactions are captured without requiring exhaustive combinatorial exploration.

### 5.10. Long-Term Robustness and Model Maintenance Strategy

The deployed HydroNeuro model operates as a static, offline-trained predictor; however, long-term robustness is ensured through an event-driven maintenance strategy designed to handle sensor drift and evolving hydraulic conditions. The residual-based anomaly detection module is continuously monitored not only for fault identification but also as an indicator of model validity. A sustained increase in residual error, bias, or false alarm rate is interpreted as evidence of distribution shift.

When such conditions are detected, a maintenance cycle is triggered, consisting of (i) sensor recalibration using a stable hydraulic reference state to compensate for measurement drift and (ii) offline model retraining using an updated dataset reflecting the current system dynamics. This strategy avoids the complexity and resource overhead of continuous online learning while preserving deterministic execution and real-time reliability on the ESP32 platform.

Additionally, periodic or sliding-window statistical updates can be incorporated to further enhance robustness in highly dynamic environments. Overall, this approach provides a practical and energy-efficient solution for maintaining long-term accuracy in real-world deployments without compromising embedded constraints.

### 5.11. Energy Efficiency Measures

The quantized 8-bit TensorFlow Lite Micro (TFLM) model exhibits exceptional memory and computational efficiency, occupying only 48 kB of flash memory and performing inference in just 8.2 ms on the ESP32 microcontroller operating at 240 MHz. Empirical power profiling using a Monsoon power monitor indicates an average energy consumption of 11.4 mW during active inference and only 0.9 mW in deep-sleep mode, assuming a 30-s sampling interval (duty cycle 0.027%). These results suggest an anticipated battery lifespan exceeding 2.5 years on a standard 18650 Li-ion cell (3500 mAh, Shenzhen Victpower Technology Co., Ltd., Shenzhen, China), corroborating the suitability of the HydroNeuro platform for long-term, autonomous deployment in remote agricultural environments.

### 5.12. Main Contributions

The main contributions of this work can be summarized as follows:

- A data-efficient framework for hydraulic anomaly detection that minimizes the need for extensive physical data collection.
- An optimized experimental strategy based on fractional factorial design (FFD) to maximize the information content of the dataset while reducing the number of required experiments.
- A hybrid domain-aware learning approach that leverages hydraulic principles to ensure physical consistency in the collected data and the learned representations.
- An end-to-end Edge-AI implementation deployed on an ESP32 microcontroller using TensorFlow Lite for Microcontrollers, enabling real-time anomaly detection under strict hardware constraints.
- Experimental validation demonstrating that reliable anomaly detection performance can be achieved with a limited dataset generated through optimized experimental design.

## 6. Discussion

We evaluate the performance and practical relevance of the **HydroNeuro** framework as a robust, embedded-AI platform for intelligent hydraulic management in agricultural networks. Unlike conventional irrigation controllers that mainly focus on scheduling or localized sensing, we extend the scope toward a holistic representation of hydraulic behavior, including water distribution, pressure regulation, and real-time anomaly detection within interconnected infrastructures [5,30,31].

By combining domain knowledge of hydraulic principles with data-driven neural inference, we provide a black-box predictive model capable of estimating and interpreting pressure–flow dynamics with high accuracy. While the MLP itself does not explicitly incorporate physical equations, the design and interpretation of results respect hydraulically plausible ranges, guided by Bernoulli’s principle, continuity laws, and Darcy–Weisbach head-loss considerations [19,20]. This approach ensures that predictions remain consistent with expected hydraulic behavior without requiring physics-informed training.

The use of a fractional factorial design (FFD) allows us to reduce the experimental burden while preserving statistical rigor. Considering the exponential number of valve combinations in multi-branch networks, exhaustive sampling would be prohibitively expensive in terms of time, water, and energy. The FFD method enables systematic identification of influential valves and interaction patterns [22], producing a compact yet informative dataset. This approach aligns with sustainability-oriented engineering practices and strengthens the scientific rigor of our model calibration [3].

For embedded deployment, we quantize and convert the neural network into TensorFlow Lite Micro and execute it directly on the ESP32 edge platform, enabling **on-device real-time inference**. We detect anomalies, including leaks, obstructions, and irregular valve behavior, without relying on cloud computation. The combination of LoRa for long-range, low-power communication and Wi-Fi activation restricted to OTA updates provides an optimal balance between operational efficiency, communication resilience, and energy management [15,17].

Our comparative modeling results confirm that the MLP significantly outperforms the linear regression baseline. We observe superior accuracy, robustness to sensor noise, and the ability to capture nonlinear hydraulic dynamics—essential attributes for real-time anomaly detection in irrigation systems [8,26]. Across both controlled and practical scenarios, HydroNeuro maintains high precision and recall in anomaly identification, validating the reliability of our embedded inference pipeline.

**Limitations.** Despite its strengths, HydroNeuro has some limitations. First, the training dataset is generated from a prototype network, which may not fully capture the diversity of large-scale or highly heterogeneous irrigation systems. Second, the current MLP model is static; it does not adapt online to long-term changes in hydraulic behavior or sensor drift. Third, while our edge implementation reduces dependence on cloud connectivity, it is constrained by the ESP32’s memory and processing resources, which may limit scalability to very large networks.

**Future Work.** We plan to address these limitations through adaptive retraining and online learning to support network evolution over time. We also aim to investigate federated learning strategies across multiple irrigation sites to enable collaborative model improvement without centralized data collection. Finally, we will explore integration with energy harvesting and predictive maintenance modules to enhance autonomy and long-term operational sustainability.

Overall, HydroNeuro demonstrates the feasibility of combining data-driven neural inference, statistical experimental design, and hydraulic domain knowledge to deliver a practical, energy-efficient, and reliable hydraulic monitoring solution. By unifying experimentation, edge AI, and domain-aware interpretation, we provide a foundation for sustainable, intelligent irrigation and a broader transformation toward self-adaptive agricultural hydraulics [4,6,32]. These results confirm that the proposed data-efficient strategy enables the training of a reliable model with a relatively small dataset, highlighting the effectiveness of combining experimental design optimization with lightweight neural inference.

## 7. Conclusions

This paper introduced HydroNeuro, an embedded-AI framework for intelligent hydraulic monitoring in agricultural irrigation networks. By integrating structured experimental design, statistical modeling, and neural inference, the proposed system enables accurate and real-time detection of hydraulic anomalies, including leaks, obstructions, and abnormal flow conditions.

The framework operates efficiently on resource-constrained ESP32 hardware, where quantized TensorFlow Lite Micro models support on-device inference with low memory footprint and minimal power consumption. Combined with LoRa-based communication, the system enables resilient and scalable deployment in distributed agricultural environments where connectivity and energy resources are limited.

Experimental validation demonstrates that the integration of fractional factorial design (FFD) with Hamming weight-based configuration augmentation enhances the representativeness of the dataset and improves model generalization. These results confirm that reliable anomaly detection can be achieved even with a relatively small dataset when experimental configurations are carefully designed to maximize informational diversity while minimizing redundant measurements.

An important outcome of this work is the demonstration that reliable AI-based anomaly detection can be achieved with a limited dataset when domain knowledge and optimized experimental design are combined. This finding highlights the potential of data-efficient AI strategies for physical systems, particularly in hydraulic infrastructures where large-scale data collection may be costly, time-consuming, or environmentally undesirable.

Future work will focus on deploying the proposed system in real agricultural irrigation networks to evaluate its performance under operational field conditions. These large-scale experiments will allow us to assess the scalability of the architecture, identify practical deployment constraints, and further refine the system to address the limitations observed in real-world installations.

## Figures and Tables

**Table 1:** Summary of the experimental design (13 configurations).

Cfg	V1	V2	V3	V4	*P*	Stage
1	0	0	1	1	0.975	FFD
2	0	1	0	1	0.997	FFD
3	0	1	1	0	1.003	FFD
4	1	0	0	1	0.993	FFD
5	1	0	1	0	0.991	FFD
6	1	1	0	0	1.004	FFD
7	1	1	1	1	0.364	FFD
8	0	1	1	1	0.531	FFD
9	1	0	0	0	5.220	HW
10	0	1	0	0	4.875	HW
11	0	0	1	0	5.385	HW
12	0	0	0	1	5.577	HW
13	1	1	1	0	0.530	BC

**Table 2:** Summary of the dataset construction and augmentation pipeline. The 13 operational states comprise 8 fractional factorial configurations, 4 Hamming weight single-valve states, and 1 empirically validated high-activation boundary state (v=[1,1,1,0]). Each configuration was measured three times, yielding 39 physical ground-truth samples. The final training set of 1339 samples combines 39 physical measurements with 1300 state-aware synthetic samples generated via adaptive Gaussian jittering with 3σ clamping.

Parameter	Value	Description
Number of valves	4	Controllable binary actuators
FFD configurations	8	$2^{4-1}$ half-fraction, Resolution IV
Hamming weight states	4	Single-valve activation (${∥v∥}_{1}=1$)
High-activation state	1	v=[1,1,1,0]; empirically validated
Unique valve states *S*	13	Total distinct hydraulic operating states
Repetitions per state	3	Independent measurements per configuration
Raw samples $N_{\mathrm{raw}}$	39	13×3 physical ground-truth measurements
Augmentation factor	100	Synthetic samples per state
Noise profile	Gaussian	Adaptive, state-aware ($\sigma_{\mathrm{eff},j}$, empirical branch for all states)
Outlier control	3σ clamp	Eliminates non-physical outliers
Total samples $N_{\mathrm{total}}$	1339	39 + 1300 (physical + synthetic)

**Table 3:** Comparative performance metrics of the linear regression baseline and the proposed MLP model on the HydroNeuro testbed.

Model	MSE (Bar^2^)	RMSE (Bar)	MAE (Bar)	R^2^
Multiple Linear Regression	0.336	0.58	0.41	0.787
MLP (proposed)	0.0144	0.12	0.08	0.999

**Table 4:** Predictive performance of the three additional candidate architectures on the held-out test set (267 samples, 20% stratified split). MSE is expressed in bar^2^; RMSE and MAE are expressed in bar. The Custom Score is defined in Equation (19). Results for the MLP and the linear baseline are fully reported in Table 3 and are not reproduced here to avoid redundancy.

Model	MSE (Bar^2^)	RMSE (Bar)	MAE (Bar)	*R* ^2^	Custom Score
Decision Tree	0.000373	0.019	0.011	0.9999	0.967
Random Forest	0.000373	0.019	0.011	0.9999	0.967
1D-CNN	0.000493	0.022	0.015	0.9999	0.962

**Table 5:** Unified summary of predictive performance and embedded deployment feasibility for all five evaluated models. Accuracy metrics for the MLP and linear baseline are sourced from Table 3; metrics for Decision Tree, Random Forest, and 1D-CNN are sourced from Table 4. ✓ denotes full architectural coherence with the ESP32/TFLM firmware pipeline; “Partial” indicates that deployment is technically feasible but introduces firmware engineering drawbacks incompatible with the design constraints of this work.

Model	RMSE (Bar)	*R* ^2^	Deployable	Firmware Structure
MLP (proposed)	0.12	0.999	✓	Compact passive byte array (TFLM)
Decision Tree	0.019	0.9999	Partial	Single branching function body
Random Forest	0.019	0.9999	Partial	100 replicated branching functions
1D-CNN	0.022	0.9999	Partial	Higher compute overhead, no accuracy gain
Linear	0.58	0.787	✓	Analytical form (accuracy insufficient)

**Table 6:** Detection performance of the HydroNeuro system under different **hydraulic scenarios**.

Scenario Rate	Accuracy	Precision	Recall	F1-Score	False Alarm
Normal operation	99.2%	99.5%	99.0%	99.2%	0.5%
Leak simulation	97.8%	98.1%	97.5%	97.8%	1.2%
Obstruction simulation	96.4%	95.8%	97.3%	96.5%	2.1%
Overall (noisy data)	97.8%	97.8%	97.9%	97.8%	1.6%

**Table 7:** Typical configurations for two-level fractional factorial designs of the form $2^{k-p}$.

*k*	Design	Runs	*p*	Fraction	Resolution	Comment
4	$2^{4-1}$	8	1	1/2	IV	Very good
5	$2^{5-2}$	8	2	1/4	IV	Common
6	$2^{6-2}$	16	2	1/4	IV	Balanced
7	$2^{7-3}$	16	3	1/8	IV	Screening
8	$2^{8-4}$	16	4	1/16	IV	Widely used
9	$2^{9-5}$	16	5	1/32	III/IV	Heavy screening
10	$2^{10-5}$	32	5	1/32	IV	Optimal trade-off
11	$2^{11-5}$	64	5	1/32	IV	Good
12	$2^{12-6}$/$2^{12-8}$	64/16	6/8	1/64/1/256	IV/III	Budget-dependent
15	$2^{15-11}$	16	11	1/2048	III	Extreme screening

## Data Availability

The data supporting the findings of this study are available from the corresponding author upon reasonable request.
